# The Action of Medicines

**Published:** 1881-01

**Authors:** A. J. Howe


### THE ACTION OF MEDICINES.

BY DR. A. J. HOWE.

In venturing upon a reply to the interrogatives above announced, I will express the wish that the questions had been clearer cut and more concise. It would seem that the querist regarded vital operations as formative or constructive in their tendencies, and chemical action as decomposing and destructive in its effects upon the living organism. I shall speak from that platform at any rate, and do the best I can with the abstruse topic. If *life* were understood in its essence we could talk more understandingly about "vital operations." A plant is organic in its method of development and composition,--in its evolutions it is subject to vital laws, and feeds largely upon chemical or inorganic substances. The dissolved crystals of rock enter its rootlets as pabulum; and the chemically acting rays of the sun transform the sap into woody stem, branch, twig and leaf. The vital and the chemical operations act in conjunction, and may be called physical, yet that familiar term does not add to the lucidity of the explanation Primarily plants furnish food for animals, consequently all food, whether of vegetables or animals, comes directly or indirectly from the *chemical*, mineral, or inorganic world. All the processes of growth, repair and decay in animal life, are of a chemical nature, yet so subtle, variable, and complex, that the chemist can only arrive at the combinations approximately, or in a speculative manner. The vital interference warps all problems worked out on the chemist's slate. The elementary constituents of air are known as it enters the lungs, but its chemical character is changed before it returns.

This expired air contains less oxygen and more carbon than when inspired,—it has been subjected to the process of aeration: and the actions, reactions, and change of properties in elementary bodies or gases were not harmful, but essential to life,—a vital state is the result of a series of chemical activities.

Water, so necessary to organic continuance, is strictly a chemical product,—a combination of several elementary entities. Water in the living organism serves to keep the mineral salts in solution; and is instrumental in carrying on the processes of nutrition. Water constitutes much the largest part of animal bodies. Water is wholly inorganic, or as much so as the crystals of a granite ledge.

The chloride of sodium (common salt) is strictly a chemical compound; yet it is essential to the processes of digestion, absorption, and assimilation. Animals will not live and thrive without regular supplies of this agent. Most saline springs whose waters have the reputation of being curative in their action upon several disorders, contain chloride of sodium in greater or less proportions.

The sulphate of magnesia (Epsom salts) is another common agent of mineral origin and chemical composition which in certain diseased states of the body, acts in a *regulative* and curative manner. The medicine does not make a chemical impression, but assists in the transudation of fluids through membranes. There are certain remedies which favor secretion or the passage of fluids through glands, and are *selective* as to the glands they impress; and sulphate of magnesia seems to have an affinity, so to speak, for the mucous follicles of the intestinal canal. The specific effect is to cure dysentery. When given in large doses it produces catharsis. The agent may be so handled as to allay irritability of the bladder, and to act beneficially in other disorders of the organism.

Sulphur is another agent of the inorganic world which is regulatory of a part of the animal economy. Probably it takes on several combinations in the alimentary canal, yet in all of its forms it exerts a beneficial action upon digestion and hepatic functions. It is one of the few agents that favorably impress the liver, and never irritates the organ. Sulphur produces pasty evacuations from the bowels, and tends to the cure of hemorrhoids. In the treatment of indigestion and constipation sulphur has no equal. The agent has been employed to advantage in the management of several varieties of skin disease.

While it is unquestionable that air, water, salt, magnesia, sulphur, iron, phosphorus, and several other mineral agencies are essential to health; and that some of the chemical changes these inorganic substances undergo are vital and conservative so far as life is concerned, the partisan Botanic might claim that these agents were natural elaborations, and not the products of the chemist's laboratory. The champion of purely vegetable medicines might argue that the bran of unbolted flour contains iron, sulphur, lime, and other substances of mineral origin found in the blood or solid tissues of the body, therefore our food furnishes enough of such elements for all practical purposes, and no necessity exists for the administration of them as medicines. While there is a smattering of truth in the argument, the experienced theraputist has met with disordered states in which ferruginous preparations could be administered to advantage. The phosphates have become almost indispensable in the management of certain morbid states.

Although Iodine is of vegetable origin, the iodides so commonly employed in medical practice are strictly chemical in their composition, and to an appreciable extent they behave chemically when brought in contact with the fluids and solids of the body. The iodides promote active tissue change, and are therefore called *alteratives.* The iodide of potassium cures neuralgia by assisting in the elimination of materials in the body which irritate the nerves; in acting upon the glandular system and thereby promoting absorption, they assist in the discussion of tumors and in the removal of secondary deposits. In thus acting the iodides do not behave as chemicals, or prove curative by chemical reactions, but by arousing certain vital activities that have become dormant. Notwithstanding what has thus far been said about minerals and chemicals, I will state that, according to my observations, readings, and reasonings, most medicines act by making impressions upon the nervous system, whether the agents be of mineral, vegetable, or animal origin. It is said that the poison of serpents is of an acid nature; well, granting that it is, who believes that enough of an alkali to neutralize the acid of the poison would render it harmless? When we speak of the sulphate of quinia, it is not for a moment supposed that the chemical addition of enough sulphur and acid to render it a sulphate, in any appreciable degree modifies what pure quinia would do. The form is one convenient for the manufacturer in the elaboration of the crystalline principle of the bark. The same may be said in regard to the sulphate of morphia, and other well known chemical forms in medicine. In general language it may be said that all medicines which act in harmony with the vital functions, do so by directly impressing the nervous system. In parturition, ergot is administered to excite activity in the muscular tissue of the uterus. The medicinal impression is not made directly upon the muscle of the organ, but on the ganglionic nerve centres which preside over uterine contractions. The medicine does not awaken energies in the muscular fibres of other organs, but confines its activities to nerves that preside over the womb. In other words, the remedial agent acts specifically and directly upon the uterus. There is no chemical agency in the operation. But when chloral is administered to subdue pain in the womb or any other organ of the body, its impression is that of a general sedative; and such is the action of chemicals as a whole, — they exert their action in a general way, and are rarely specifics.

In reviewing the subject of remedial action upon the living organism we find few agents that antidote animal poisons, yet many agents are named which promote elimination of morbid matter, and others which help in the work of reparation or in hastening constructive metamorphosis, — in the elaboration and assimilation of nutrients. A gouty poison in the digital articulations cannot be antidoted *in situ*, but it may be reached through eliminative action. The object of the physician should be to administer such medicines as, by acting upon the kidneys and other emunctories, will drain the blood of its poisons, and thereby prevent additional deposits in the joints. As soon as the supply is cut off, the quantity on hand will soon disappear. If the source of production be in the stomach, as it often is, the use of peptics will cure the gout. There is no objection to the use of colchicum and other depuratives, yet as the stomach is the laboratory of gouty supplies, the cure is to commence there. The patient may suffer from acid eructations and complain of "sour stomach," but the physician is not to infer that bicarbonate of soda or other alkaline agents are indicated. Nux and lactopeptine will generally do much towards curing gout, especially if easily digested foods be included in the rational treatment.

Not unfrequently an excess of heat is the chief source of danger in certain diseases. In acute meningitis and cerebritis the blood is propelled with violence to the head, raising the temperature six or eight degrees above its natural standard, — the source of peril is in the excessive heat, — and what may be done to lower the temperature? Shall we rely upon the action of arterial and cardiac sedatives, or can greater progress be made by evaporating lotions? Cooling applications to the head will accomplish a greater reduction of heat in five minutes than internal medicines would in five hours, — and a fatal lesion might occur in that time. Mr. Spencer Wells applies ice to the heads of those women on whom he observes a high degree of heat after ovariotomy; and he thus lowers the temperature at once. In cases of brain fever I apply a dilute tincture of aconite to the scalp, and repeat the operation every few minutes. If this treatment be begun early and kept up faithfully, there will be no damaging effusions of lymph into the cerebro meningeal spaces. No internal medicine will do any appreciable good; but the patient is to be fed with gruel, beef tea, and ice cream. The evaporation of the lotion lowers the temperature, and aconite sedates the irritated nerves.

In the treatment of malarial intermittent fever there is no agent so potent in the exhibition of its antiperiodical powers as quinia. The medicine is an acknowledged specific in the management of miasmatic diseases; and it exerts its peculiar influence upon the nervous centers. There is no chemistry in the matter, but a purely antidotal action of a physiological character. Quinia exerts other beneficial impressions upon morbid phenomena, yet in none so prominently as in malarial fever. The daily chills of tuberculous and wasting diseases are better overcome by the use of Fowler's Solution of arsenic. A drop of the medicine is a dose that may be repeated several times a day. Although a chemical compound, the action of the medicine is not of a chemical nature. The agent seems to arouse the activity of the digestive and recuperative functions.

In the treatment of a morbid feature that goes by the name of "night sweats" we have several agents that exert a restraining influence over exhaustive perspirations. Cold sage tea is the simplest of the lot, and often makes all the favorable impression that is needed,—in other words, it is quite commonly sufficient. Elixir vitriol also answers a good purpose, as well as oxide of zinc. It is not improbable that the astringency of the latter two has something to do with the remedial effect, yet other astringent medicines do very little good in restraining night sweats. Certainly there is not enough astringency in sage to check a profuse sweat that attends sound sleep,—the agent must make a specific impression upon the perspiratory functions, the nerves being the first to receive the restraining influence. But the most potent agent to check sweats is atropia. The one hundredth of a grain of sulphate of atropia, repeated every six or eight hours if necessary, is almost a sure cure for nocturnal sweats. The medicine does not act as a re-agent in any rational sense, but directly, through the nervous system.

Santonine is a medicine administered to destroy certain classes of intestinal parasites, and is to that extent valuable. The agent in efficient doses produces no unpleasant effect upon the patient, yet exerts a destructive influence upon intestinal worms. Pomegranate bark will destroy tape worm, yet in a way not well understood. We could not say that the medicine does anything more than to act as a poison to the parasite. It is even probable that some varieties of the tænia will resist the poisonous effects of pomegranate.

Apocynum is a medicinal agent which produces watery evacuations of the bowels, and profuse excretions of urine, hence it is one of the most valuable of remedies that can be employed in the treatment of ascites and general dropsies. When dropsical effusions depend upon cardiac disorders the use of digitalis should be employed in alternation with apocynum.

It is adding weight of evidence to the argument that medicines act by impressing the nervous system, to array on that side the specific action of belladonna in dilating the pupil of the eye: and in the same connection the fact that opium and calibar bean will cause the pupil to contract. It could not be said that these agents have an affinity for the nerves that excite the muscular apparatus of the iris, yet it would be difficult to show in just what way they do exert these peculiar influences upon the pupil.

Veratrum acts directly and almost specifically upon the ganglionic nerves which preside over respiration and the circulatory functions. It is the best febrifuge in existence; and its effects upon the cerebro-spinal system of nerves are antagonistic to convulsive action. The medicine also acts favorably upon mesenteric glands that have been obstructed by tuberculosis. When given in connection with arsenic to persons suffering from incipient phthisis the recuperative impression is prompt and marked.

Strychnia is a medicinal agent of value in treating atony of the stomach. It must be given in minute doses, for the dangers in poisonous quantities are of a character to be shunned. The agent in toxic doses, produces spasmodic jerking of the muscles, and brings about death by asphyxia. The impression on the nervous system is apparently not unlike that which produces tetanus.

Alcohol is rather peculiar in its effects upon the organism. Its action is directly upon the brain, exciting mental activity and awakening pleasant thoughts. In considerable quantities the agent produces delirious excitement, perverting the judgment and the moral nature. The secondary effects are those of stupor and intellectual hebetude. While alcohol is exceedingly useful in the manufacture of drugs; and is a prominent ingredient of brandy, whisky, rum, gin, wine, beer, and cider, yet the agent is rarely needed as a stimulant in the management of the sick, unless it be to tide a patient over a crisis or a temporary state of depression.

Opium is a drug that sedates the cerebro-spinal centres, and retards functional activity in all parts of the body. Like alcohol it exhilerates at first and stupefies afterwards, yet with less of the earlier action and more of the latter. The opiates are exceedingly valuable in assuaging pain. The quieting effect is profound and prolonged. A minute quantity of morphia is excellent to allay a troublesome cough. It is thought by many physicians that there is nothing curative in the influence of an opiate, but when pain and irritation are rapidly wearing out the machinery of the body and the causes of the friction can not be removed, the soothing effect of an anodyne is invaluable. Pain will kill, therefore it should be assuaged if practicable.

Chloroform and sulphuric ether are chemical compounds that directly impress the nervous system, and in their action behave much like alcohol. Anæsthesia is only drunkenness produced by inhalation of ethereal narcotics. These agents by their exciting impressions at first hasten vital activities, and at length beget stupor and a state of unconsciousness.

Inasmuch as all of the medicinal agencies named are valuable as remedies if the dose be proper, and the therapeutical indication well selected, it may be said that their action is in harmony with vital operations, — is *physiological*, — yet who is wise enough to always see the special applicability of a particular medicament to variable and varying morbid states? In fact, no mental scope is comprehensive enough to embrace a subject which admits of endless variations. A medicine that is for the time adapted to a given morbid condition may do damage the next minute when the symptoms have changed. It seems that the only safe way is to medicate so restrictedly or guardedly that no substantial harm can be done though the once timely remedy has become unsuited by circumstances. Medicines which assist the vital processes to restore a deranged function or disordered state, are valuable whether they come from the mineral, vegetable, or animal world, or be chemically elaborated from elementary or compound bodies. Remedies coming from the vegetable kingdom, if employed at the right time and in proper doses, generally act in harmony with vital forces, yet the rule is not so sweeping that mineral agencies can be safely neglected. In dealing with these subjects the main point is to keep clear of prejudice and preconceived notions. If a practitioner has made up his mind without experimental knowledge that arsenic, for instance, is a poison, and therefore should not be used as a medicine, he has allowed his mental faculties to be bound with fetters, although he may at the same time boast of his independence and liberality. There is nothing easier than to be mistaken about one's self.

In nineteen cases of sickness out of twenty the recuperative forces of the body are able to bring about a state of health, therefore the physician who does nothing or no harm will meet with excellent success; and if he prescribes certain agents with little or no medicinal power, he is apt to ascribe all the cures to the medicine, and nothing to the restorative functions of the body. The therapeutist is to consider that in sickness he is to help the vital activities in the work of repair — to "assist Nature." The expression is old and homely, yet expressive of truth. He is to see that the remedial agencies brought into service shall act in harmony with the "regulative" forces of the nervous system. Sometimes it is so difficult to see what kind of aid Nature needs, that only symptoms can be safely treated: and if these be carefully watched and skillfully weighed, the importance of the morbid process may be comprehended. However, when we name a disease from observing symptoms we go from effects to cause, which is a very uncertain and unsatisfactory method of reasoning. In fact, this is the avowed Homœopathic process of arriving at conclusions in therapeutics, and one which is suited to an expectant or placebo plan of treatment. Symptoms are in view when medicine is given, and not the disturbing element, or the cause of disordered action.—*Eclectic M. D. Journal.*

